# How developmental neuroscience can help address the problem of child poverty

**DOI:** 10.1017/S0954579420001145

**Published:** 2020-12

**Authors:** Seth D. Pollak, Barbara L. Wolfe

**Affiliations:** 1 Departments of Psychology and Waisman Center, University of Wisconsin, Madison, WI, USA; 2 Departments of Economics, Population Health Sciences and Public Affairs, University of Wisconsin, Madison, WI, USA

**Keywords:** brain, child poverty, development, socioeconomic status

## Abstract

Nearly 1 in 5 children in the United States lives in a household whose income is below the official federal poverty line, and more than 40% of children live in poor or near-poor households. Research on the effects of poverty on children’s development has been a focus of study for many decades and is now increasing as we accumulate more evidence about the implications of poverty. The American Academy of Pediatrics recently added “Poverty and Child Health” to its *Agenda for Children* to recognize what has now been established as broad and enduring effects of poverty on child development. A recent addition to the field has been the application of neuroscience-based methods. Various techniques including neuroimaging, neuroendocrinology, cognitive psychophysiology, and epigenetics are beginning to document ways in which early experiences of living in poverty affect infant brain development. We discuss whether there are truly worthwhile reasons for adding neuroscience and related biological methods to study child poverty, and how might these perspectives help guide developmentally based and targeted interventions and policies for these children and their families.

In 2013, the American Academy of Pediatrics added “Poverty and Child Health” to its *Agenda for Children* (American Academy of Pediatrics, 2013) as a recognition of the broad and enduring effects of poverty on children’s development. These public health implications are so profound that both UNICEF (United Nations International Children’s Emergency Fund) the World Bank have not only recognized the serious problems caused by child poverty, they have also called for the need to end extreme poverty by 2030 (UNICEF and World Bank Group, 2016). Children living in poverty are more likely to have poor health compared to peers not living in poverty, and this gap in health widens as children age (Case et al., 2002; Fletcher & Wolfe, 2014). Children from impoverished families do worse on nearly all measures of academic attainment, from school readiness to grades to standardized test scores (Duncan & Murnane, 2011; McKinney, 2014; Schüetz, Ursrpung & Woessman 2005). Compared to children in financially-secure settings, children in poverty have high rates of behavioral problems (Ackerman, Brown & Izard, 2004; Brooks-Gunn & Duncan, 1997; Duncan, Brooks-Gunn & Klebanov, 1994). These developmental gaps persist into adulthood and are reflected in lower lifetime earnings, worse health, and reduced psychological well-being (Al Hazzouri, Haan, Galea & Aiello, 2011; Minkler, Fuller-Thomson & Guralnik, 2006; Wadsworth et al., 2016). Although the associations between child poverty and negative outcomes are well documented, the mechanisms causing these sequelae are not well understood. A relatively recent addition to the field has been the application of brain-based methods to better understand the developmental consequences of child poverty. Here, we address questions about whether and how these approaches might be useful in guiding developmentally based and targeted interventions and policies for children living in poverty.

## Setting the Context: What do we Mean by Poverty and Socioeconomic Status?

It is often difficult to compare studies on the effects of poverty on child development. This is because of the wide and inconsistent range of variables that researchers use to define their samples (Pollak & Wolfe, In press). As we will explain below, among researchers there is no single measure of what constitutes poverty. A second issue is a lack of clarity between a family’s income and their socio-economic status (SES) (Farah, 2018). Simply put, poverty reflects low income or low access to resources. SES is an index of who is better off or worse off in a given society. Although often used interchangeably, these are different constructs. For example, the amount of money someone makes is not the same as occupational prestige. A graduate student may have a very low income in the short term but will eventually have a high income; a minister may have a low income but also free housing and high local prestige. For these reasons, understanding how family circumstances are affecting the development of children may require that researchers distinguish factors such as higher versus lower income, more versus less education, better versus worse neighborhoods, and prestigious versus less prestigious jobs. While these factors are associated with each other, they each confer slightly different risk and protective factors for children.

Operationally defining poverty – especially in a global context – is a complex issue (Pollak & Wolfe, In press). Much has been published about what constitutes poverty, how to define it, and how to measure it (Institute for Research on Poverty, 2016; Short, 2016). Issues range from whether to include only income or also in-kind benefits; the length of time under consideration (because families can move in and out of poverty over different periods of a child’s life, depending on how poverty is measured); whether poverty measures should be absolute or relative to the median income in a given community; whether poverty measures should give an indication of the depth of poverty and whether a measure of child poverty should go beyond family income to include broader factors such as parent’s human capital and/or social isolation.

Studies of child poverty in the United States often make reference to a threshold called the Federal Poverty Line (FPL). This concept was developed by Mollie Orshansky of the Social Security Administration in the 1960s (Watts, https://www.irp.wisc.edu/publications/focus/pdfs/foc92e.pdf). The FPL is updated each year by the Census Bureau. The Census Bureau uses a set of money income thresholds that vary by family size and composition to determine who is in poverty. If a family’s total income is less than the family’s threshold, then that family and every individual in it is considered in poverty. The official poverty thresholds do not vary geographically, except for Hawaii and Alaska; they are updated annually for inflation using the Consumer Price Index. The official poverty definition uses money income before taxes and does not include capital gains or noncash benefits (such as public housing, Medicaid, and SNAP, the Supplemental Nutrition Assistance Program). This threshold was initially developed to provide a yardstick for progress or regress in government anti-poverty efforts, but it is important for child development researchers to recognize that the FPL is a simplification of the phenomenon of poverty created for administrative uses, such as determining financial eligibility for certain federal programs.

The FPL is used to determine who is eligible for certain federal subsidies and aid such as Medicaid, SNAP, Family and Planning Services, the Children’s Health Insurance Program (CHIP), the National School Lunch Program, and subsidies on the ACA (Affordable Care Act) exchanges. The actual percentage of the FPL determining eligibility may sometimes be set by States so long as they are within parameters set by the federal government. Thus, the FPL is not meant to be an index of what people need to live well or to allow children to thrive, and it is not clear that an income above the FPL is sufficient to support a family with young children. Research suggests that families with children need an income of at least twice the FPL to meet most basic needs, on average and varying by location (Cauthen & Fass, 2008). Part of the reason for this misalignment is that the original FPL was based on the premise that food accounted for a third of a low-income family’s expenditures, but that is less true today.

The issue of a family’s local cost of living does not factor into the FPL, but creates significant variance. As an example, in 2019 the federal government classified a family of four earning up to $117,400 as low-income in the San Francisco Bay Area (https://www.huduser.gov/portal/datasets/il/il2018/2018summary.odn). To generate this number, officials at the Department of Housing and Urban Development factor in the median income and average housing costs in an area (an index slightly different from the FPL). For reference, an annual income of between two and seven times the California Poverty Measure is considered middle class. By this estimate, a middle-class income in the San Francisco Bay Area would range from $74,750 to $261,623 (https://www.ppic.org/interactive/california-poverty-by-county-and-legislative-district/). By way of contrast, a family of four earning $63,600 would be classified as low-income in Champaign-Urbana, IL, and a middle-class income in this area would range from approximately $43,000 to $130,00.

There are other ways that researchers measure child poverty. Some research teams use questionnaires to target income, whereas many other researchers in Organization for Economic Co-operation and Development (OECD) countries define families living below 40% or 50% of the median income of that country. Studies of child poverty in developing countries tend to use dollars per day below a set benchmark. Still other researchers calculate an income-to-needs ratio, the concept used for the FPL, where needs are defined as the FPL. In other cases, a family’s specific situation might be referenced such as the presence of a person with significant disabilities, which is likely to increase “needs” beyond the FPL.

Other researchers focus on specific aspects of the experience of poverty, such as food insecurity, availability of stable housing, or minimum standards in housing. Recently published reports have used a vast array of different kinds of questions for research participants to characterize a child’s family as living in poverty. These range from varied and idiosyncratic ways to ask research participants about their family income, to asking about the mothers’ level of education. This latter issue of maternal education is problematic, as discussed below.

Frequently in developmental science, parent education is used as the sole proxy for children’s socio-economic environments. However, parent education, alone, provides little precision or insight into how children experience poverty (Duncan & Magnuson, 2012). Moreover, parental education is more a measure of SES, which is a different construct from poverty or family income (Pollak & Wolfe, In press). It is not yet clear whether low family income has the similar developmental effects on children as low family SES (Hackman, Farah, Meaney, 2010). A recent article that compares income and SES effects on the health of older adults finds that income is a separate and more closely tied gauge than other measures of SES (Darin-Mattsson, Fors & Kåreholt, 2017).

In summary, there is no single, simple measure of family income or parent education that is sufficient to index the developmental context of poverty for a child. Even while objective indices such as the federal poverty line may provide a useful parameter for recruiting a study sample, there is no evidence that a child living marginally above the federal poverty level is appreciably better off than one marginally below, and some researchers include those living below 133% or 200% of the FPL as poor or near poor. Moreover, poverty and SES are separate, albeit overlapping, constructs with different implications for children’s development. For these reasons, researchers need to be mindful of the fact that many measures of child poverty are limited, likely underestimate poverty, may not consider other resources available to children such as tax credits, food stamps, or subsidized housing on the positive side or tax liabilities, out-of-pocket medical costs, or work-related expenses on the negative side (each could either over or undercount resources available to children), and often make no adjustment for geographic variation. While US researchers tend to measure deprivation by assessing whether households can afford to meet a set of basic needs, many other developed countries use a “relative” measure of poverty based on the share of families below 40% or 50% of median income, on the premise that in a developed society, measuring the number of families far from the median provides a better measure of whether families are outside of the social mainstream.

## How Many Children are Affected by Poverty?

**Figure 1.**
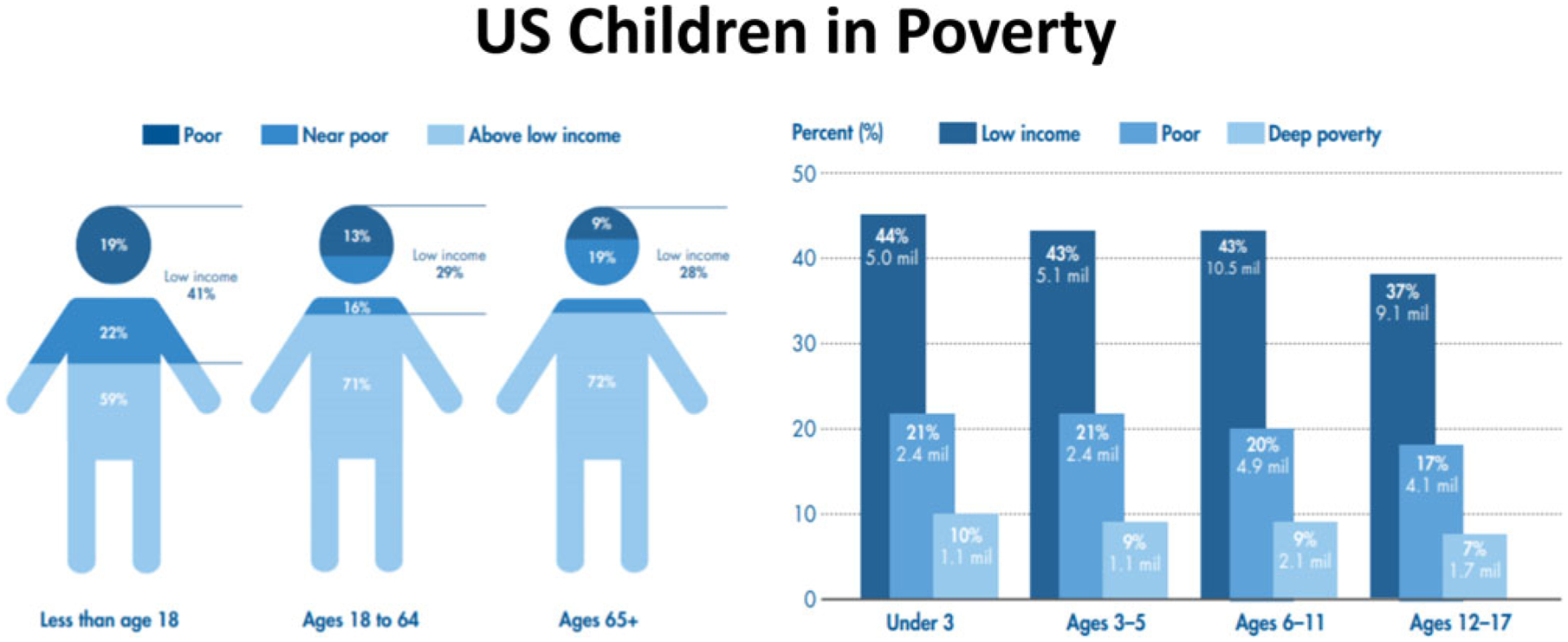
Among all children under 18 years in the United States, 41% are low-income children and 19% –approximately one in five – are poor. This means that children are overrepresented among our nation’s poor; they represent 23% of the population but comprise 32% of all people in poverty. Many more children live in families with incomes just above the poverty threshold. The percentage of low-income children under age 18 years surpasses the percentage of low-income adults. © National Center for Children in Poverty (www.nccp.org) *Basic Facts about Low-Income Children: Children under 18 Years, 2016 Reprinted with permission*

Nearly 40% of children in the United States live in poor or near-poor households (Child Trends Database, 2018; Figure 1). Specifically, in 2017, 12.8 million children in the United States were living in households with incomes below the official poverty threshold; 39% of these children were living in households with incomes below twice the poverty threshold (Child Trends Databank, 2018). These numbers reflect only a limited 12-month snapshot of child poverty. Most of these children have parents who work, but low wages or unstable employment result in insufficient family resources. The number of children in the United States who spend some portion of their childhoods living in poverty line is far higher than any single year estimate, with the youngest children at the greatest risk (Jiang, 2016). Developed countries other than the United States have lower rates of poverty, but there are still substantial numbers of children in these countries who are living in under-resourced families. According to UNICEF, among 35 economically advanced nations, the rate of children living in poverty ranged from 4.7% in Iceland, to 13.3% in Canada, 23.1% in the United States, and a high of 25.5% in Romania (UNICEF, 2012). In the developing world, UNICEF estimates that extreme child poverty (living on less than US $1.90 per day) describes 19.5% of children, compared to 9.2% of adults. This translates into approximately 385 million children living in extreme poverty across the globe (UNICEF and World Bank Group, 2016). A report by the US National Center for Children in Poverty (Koball & Jiang, 2018) reveals that 44% of children in the United States under age 9 years live in low-income families with 21% defined as poor (at the FPT) and 23% as near poor (100%–199% of the FPT); those percentages represent about 15 million children.

## What is it About Poverty that Affects Children’s Development?

From a developmental science perspective, the effects of child poverty are likely to be multidetermined (Duncan, Magnusson & Votruba-Drzal, 2017). While a full review of the poverty literature is beyond the scope of this paper, even a partial listing of candidate factors highlights the range of issues under the umbrella of “poverty” potentially affecting children. Causal factors that have been proposed to link poverty to poor outcomes in children have included limited access to medical care and insurance (Wherry & Meyer, 2016); high exposure to pollution and environmental toxins known to affect neurological functioning (Currie, Greenstone, & Moretti, 2011; Currie et al., 2014; Rowe et al., 2016); high exposure to violence (Cancian, Slack, & Lang, 2010); inadequate nutrition (de Groot et al., 2015); high exposure to infectious diseases (Hotez, 2011); social pressure associated with income inequality or low income relative to a local community (Buttrick & Oishi, 2017; Halfon, Larson, Son, Lu, & Bethell, 2017; Pickett & Wilkinson, 2015); low economic mobility (Baulch & Hoddinott, 2000; Chetty & Hendren, 2018); environments characterized by instability and chaos, as reflected in factors such as food insecurity and unstable housing (Evans & Garthwaite, 2014; Schneider, 1992); and institutionalized racism (Chetty, Hendren, Jones, & Porter, 2018) and, of course, stress which we discuss in depth below.

One reason that it is not yet clear which of these factors causes problems associated with poverty is that it might be poverty itself that is the problem. When a child lives in poverty, many of these factors are present at the same time, over a protracted period of time. Rather than any one or two of these factors being primary in influencing a child, it may well be that it is the confluence of multiple factors that threatens a child’s well-being.

## How Might New Scientific Approaches Help?

Various neuroscience techniques such as neuroimaging, neuroendocrinology, cognitive psychophysiology, and epigenetics are now being employed to examine aspects of brain development and functioning associated with early experiences of living in poverty. There are many good reasons for considering these types of biological methods alongside the traditional social science approaches to study child poverty. For example, it is well established that early experiences are critical for shaping many aspects of brain development related to children’s behavioral functioning (Birn, Roeber, & Pollak, 2017; Fox et al., 2010; Johnson, 2001; Romens, McDonald, Svaren, & Pollak, 2015; Wismer Fries, 2005). In humans, maturation of the brain regions responsible for higher cognitive functioning continues throughout childhood and adolescence, leaving a long window of opportunity and vulnerability for environments to influence brain plasticity (Blakemore et al., 2006; Bunge et al., 2002).

Traditionally, much of the research on child development in the context of poverty has focused on reduced stimulation and reduced opportunities for learning compared to children in higher-income homes (Jensen et al., 2017). However, it is not obvious how environments marked by poverty influence developmental mechanisms. For example, poverty is also characterized by an overabundance of types of stimulation that can negatively affect development. These factors include the presence of enduring stressors such as ambient noise (including background noise such as that associated with ongoing and unmonitored television), persistent household chaos, recurring conflicts among family members, exposure to environmental toxins, parental stress, and neighborhood violence – any of which might possibly alter physiologic systems involved in stress regulation, comfort, and perceived security/stability (Coley, Lynch & Kull, 2015; Deater-Deckard, Sewell, Petrill & Thompson, 2010; Evans & Kim, 2013; Hair, Hanson, Wolfe, & Pollak, 2015; Miller & Chen, 2013). Thus, there may be numerous (and not mutually exclusive) potential chronic effects on neural activity that can influence brain and behavioral development (McEwen & Gianaros, 2010; Smith & Pollak, 2020). For these reasons, the use and integration of a variety of behavioral, cognitive, and neuroscience measures permits researchers to better understand exactly how and why poverty reduces the potential of children. The addition of these biological approaches to the social science disciplines that traditionally address poverty holds tremendous promise for increasing knowledge that could lead to more effective policies aimed at reducing the negative sequelae of poverty.

Although research on poverty and brain development in humans is relatively recent, the cumulative evidence thus far is yielding new and highly convergent perspectives on how and why poverty may be linked to myriad behavioral outcomes throughout the life course. There have been a number of detailed reviews of recent findings pertaining to child poverty and the brain, so we direct readers to these recent and thorough papers rather than reproducing a full literature review here (see Blair & Raver, 2016; Johnson, Riis, & Noble, 2016; Farah, 2018).

**Figure 2.**
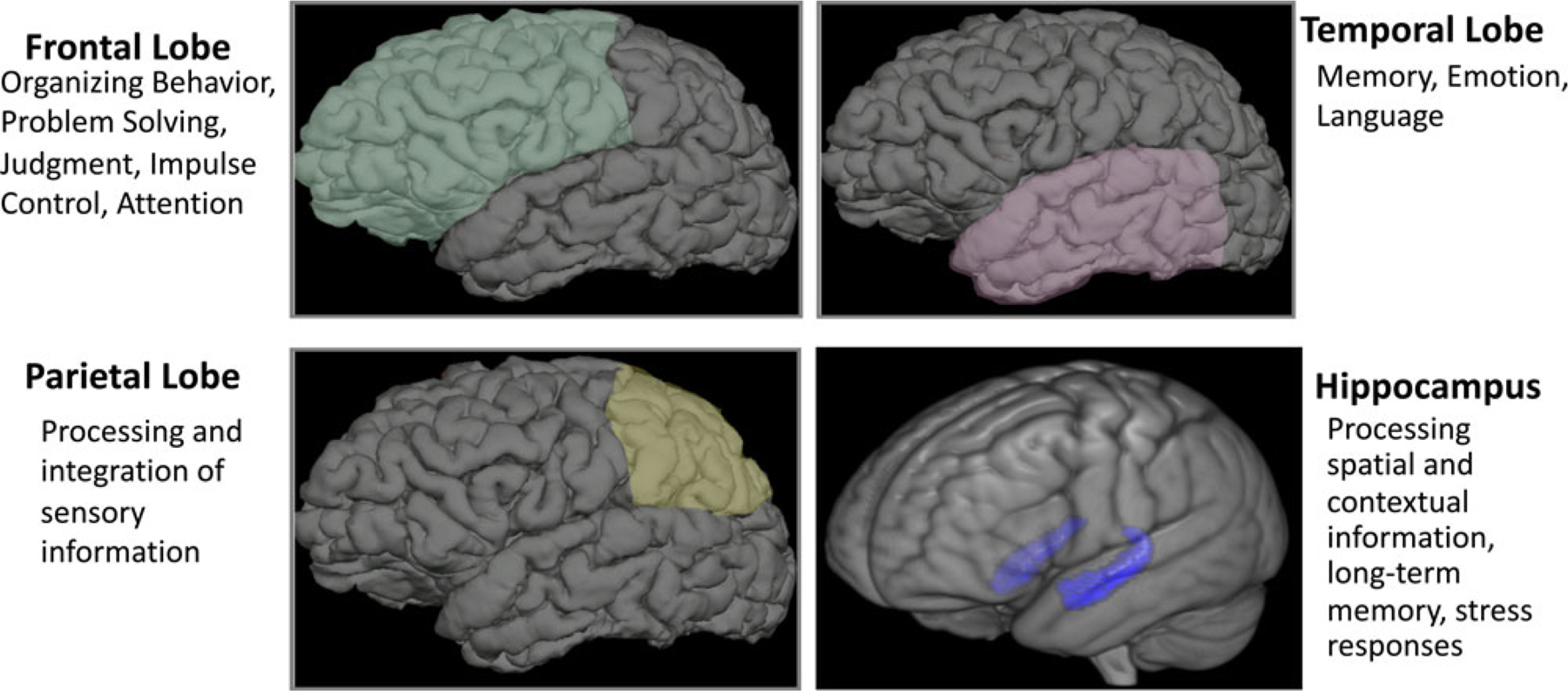
Brain regions that appear to consistently show negative associations between child poverty and gray matter development.

**Figure 3.**
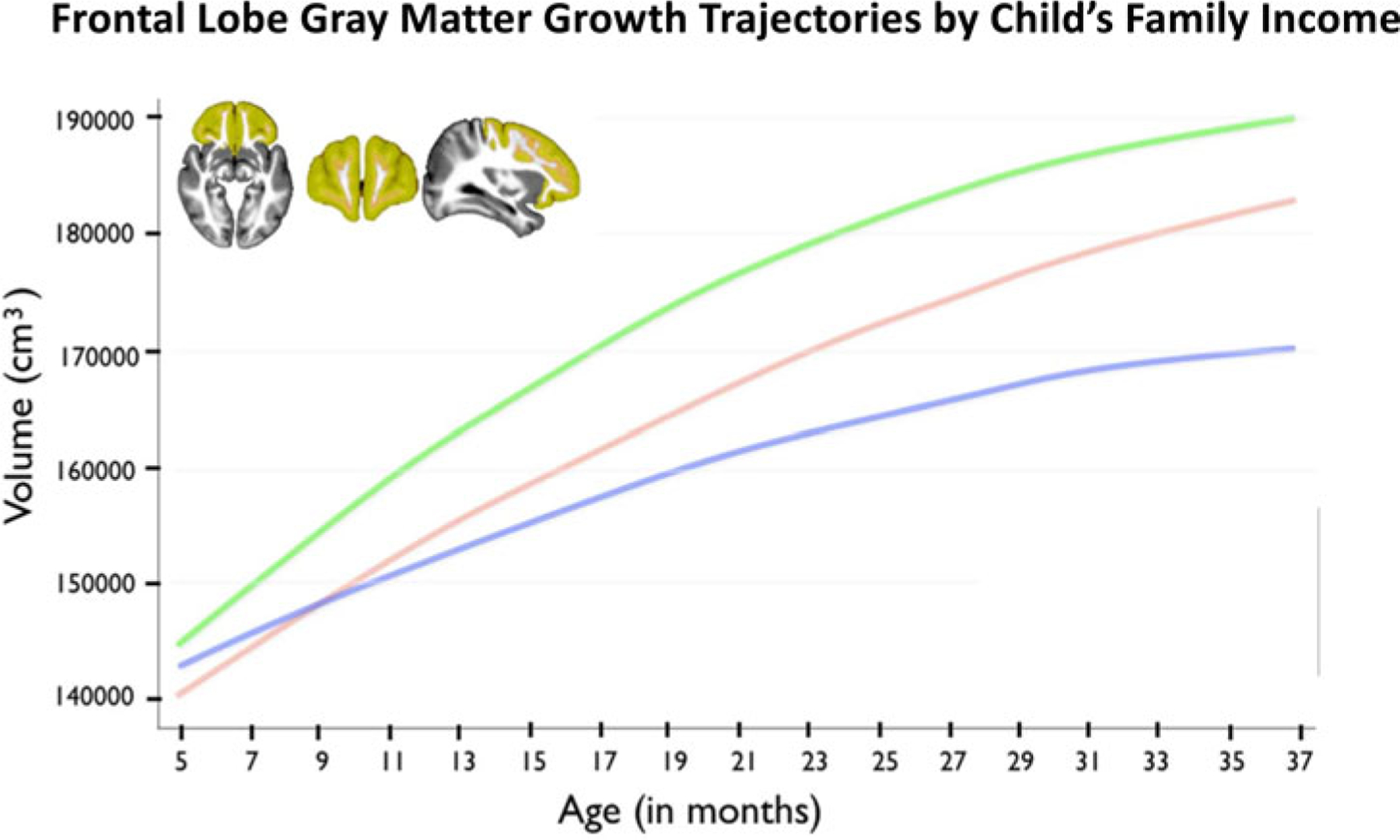
Differences in trajectory of brain growth among infants from low (blue), middle (red) and high (green) income families. There is no statistical difference between the growth rates of those from middle and high income families. Reprinted from: Hanson et al. (2013).

**Figure 4.**
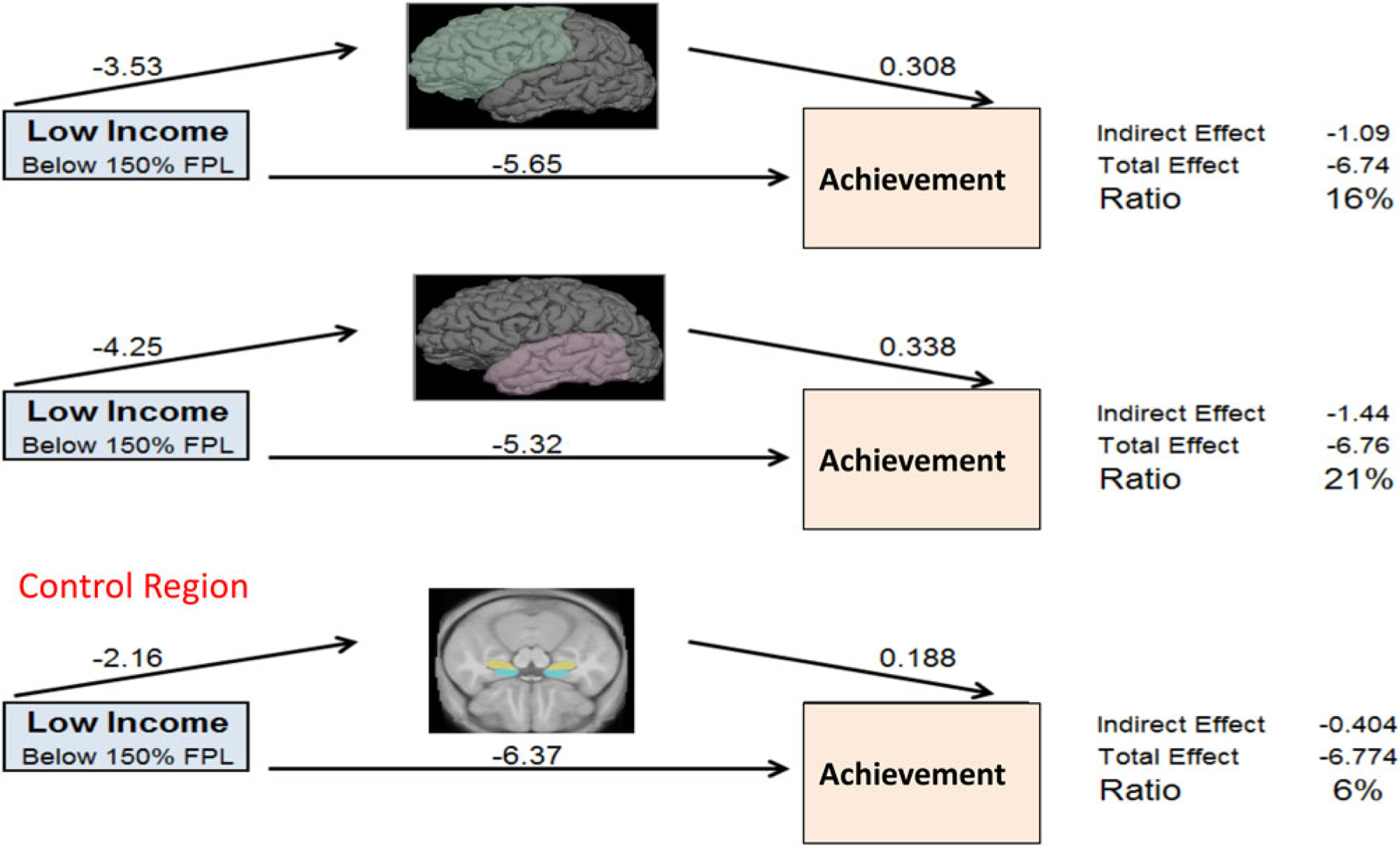
Data from Hair et al. (2015) is used to show the relationships between low family income, children’s brain growth, and children’s subsequent performance on Math Computation and Reading Comprehension achievement tests.

Our own work began by examining the effects of poverty on brain regions tied to academic functioning in children (Figure 2). We focused on brain regions known to have protracted periods of postnatal development, and brain tissue with low levels of heritability (and therefore a higher likelihood of being influenced by a child’s experiences). This included tissue such as gray matter (the parts of the brain consisting mainly of nerve cells) and brain regions including the frontal lobe (with ties to the organization of behavior, judgment, impulse control, and attention), the temporal lobe (implicated in memory, language, and emotion), and the hippocampus (associated with learning, memory and processing of contextual information). Our initial finding was an association between SES and the hippocampus, a brain region known to be affected by stress. We measured the volume of brain regions from brain images (*N* = 317) acquired from children across the socioeconomic spectrum. Children from lower-income backgrounds had lower hippocampal volumes (Hanson, Chandra, Wolfe, & Pollak, 2011). We next examined the trajectories of brain development in infants and toddlers between 5 months and 4 years of age, as children began to experience the effects of poverty. These children underwent magnetic resonance imaging (MRI) scanning, completing between 1 and 7 scans longitudinally. We found that infants from low-income families had less gray matter, tissue critical for processing of information and execution of actions. Children from lower-income households in this study had slower trajectories of brain growth during infancy and early childhood (Hanson et al., 2013; Figure 3). In a subsequent study, we examined whether these poverty-related differences in brain growth were associated with children’s academic functioning. Using a longitudinal cohort study of participants from 4 to 22 years of age, we found that poverty was tied to structural differences in several areas of the brain associated with school readiness skills, with the largest influence observed among children from the poorest households. Gray matter volumes of children below 1.5 times the federal poverty level were significantly below developmental norms. These developmental differences had consequences for children’s academic achievement. On average, children from low-income households scored lower on standardized educational tests of skills such as reading comprehension and math computation, and as much as 20% of the gap in test scores could be explained by maturational lags in development of the frontal and temporal regions of the brain (Hair et al., 2015; Figure 4).

**Figure 5.**
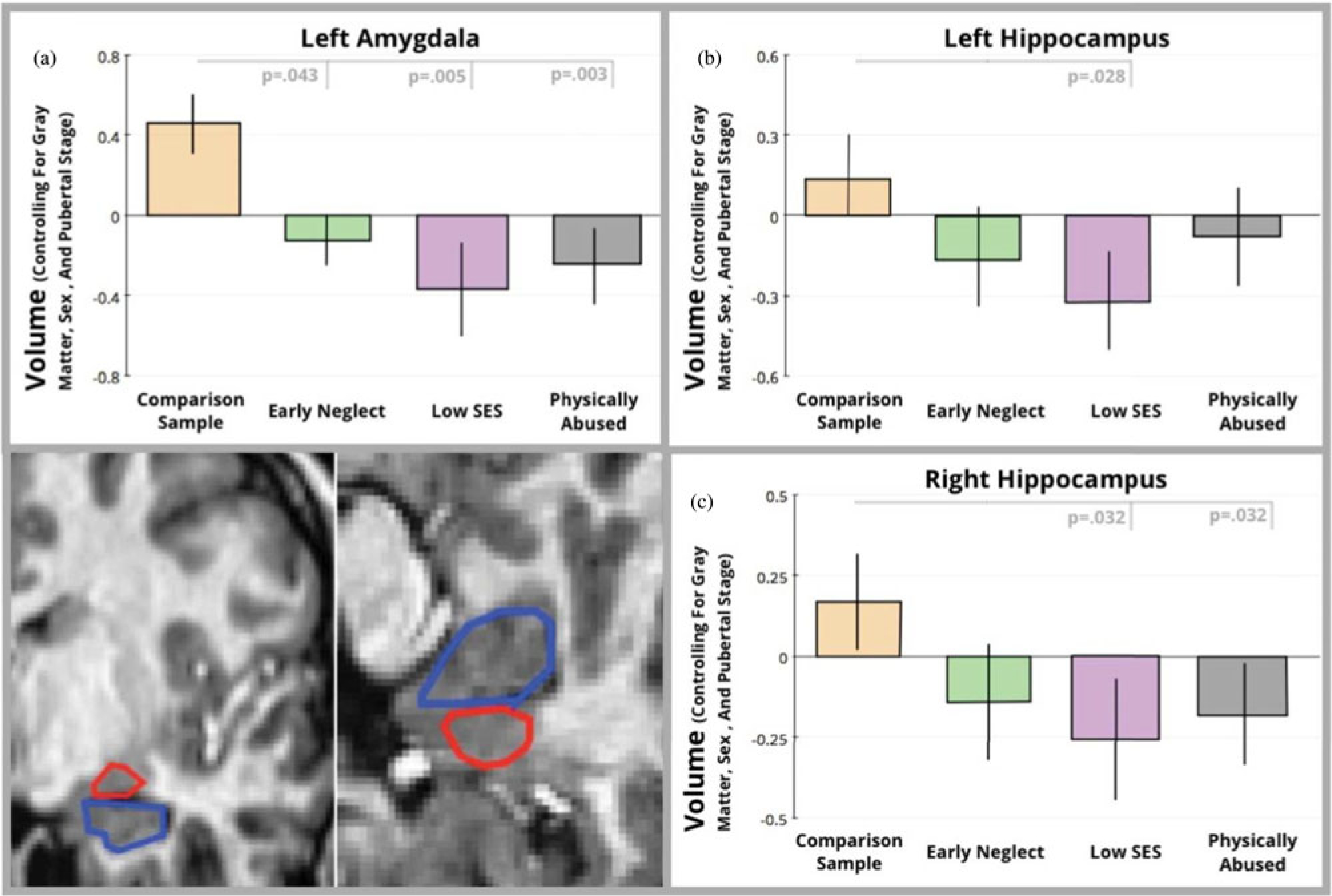
Volumetric comparisons for the left amygdala (panel a) and hippocampus (Left hippocampus shown in Panel b; Right hippocampus in Panel c). For each graph, standardized residuals controlling for total gray matter, pubertal stage, and sex are shown on the vertical axis, while group is shown on the horizontal axis. In the bottom corner of the figure are example hand-tracings of the amygdala (outlined in red) and hippocampus (outlined in blue). Reprinted from Hanson et al. (2015) with permission.

What has been notable and striking is that although the neuroscience of poverty is a relatively new and emerging area of scholarship, findings across independent laboratories, using unique samples, have been highly convergent. Most studies of the correlates of poverty have focused on regional changes in brain morphology in regions related to language, emotion, and executive functions (Brito & Noble, 2014). These include replicated associations of poverty with the hippocampus (Barch et al., 2016; Brody et al., 2017; Ellwood-Lowe et al., 2018; Hair et al., 2015; Hanson et al., 2011, 2015; Luby et al., 2013), amygdala (Brody et al., 2017; Hanson et al., 2015; Javanbakht et al., 2015; Kim et al., 2013; Luby et al., 2013; Merz, Tottenham, & Noble, 2018; Muscatell et al., 2012), and prefrontal lobe (Hair et al., 2015; Hanson et al., 2013; Holz, Laucht, & Meyer-Lindenberg, 2015; Noble et al., 2006, 2015; Figure 5). Differences also emerged for two different indices of the communication between brain regions, resting-state functional connectivity (Sripada, Swain, Evans, Welsh, & Liberzon, 2014), and white matter tracts (Dufford and Kim, 2017; Gianaros et al., 2013; Gullick et al., 2016; Noble et al., 2015). The largest sample to date reported widespread reductions in the surface area of the brain associated with childhood poverty (Noble et al., 2015). In another study, lower family income tended to be associated with reduced activation of the frontal lobe when children had to activate their memory systems (Finn et al., 2017). These differences in brain function explained differences in mathematics achievement test scores, an effect similar to our earlier (2015) findings. To date, multiple papers have reported associations between socioeconomic disadvantage and reduced cortical gray matter, as measured in terms of volume (Hair et al., 2015; Jednorog et al., 2012), thickness (Lawson, Duda, Avants, Wu, & Farah, 2013; Mackey et al., 2015), and surface area (Noble et al., 2015). These brain measures correlate with measures of language development (Romeo et al., 2018), executive functioning (Noble et al., 2015), standardized tests of academic achievement (Finn et al., 2017; Hair et al., 2015; Mackey et al., 2015), memory (Leonard, Mackey, Finn, & Gabrieli, 2015), and well-being/health (Evans, 2016; Krishnadas et al., 2013). Thus, while research in this area is still in a relatively early stage, there is a high degree in consistency among the findings.

This new focus on biobehavioral mechanisms underlying poverty is poised to guide empirically based and targeted interventions and policies for these children and their families, as well as offering promise about ways to evaluate the effectiveness of various anti-poverty programs focusing on children’s development. This is an important and timely issue given that most anti-poverty programs suffer from low effect sizes. A fairly recent review of studies that evaluate early schooling found little robust evidence of significant, positive effects of most interventions (Duncan & Magnuson, 2013). The authors found “education programs appear to boost cognitive ability and early school achievement in the short run. However, most of them show smaller impacts than those generated by the best-known programs, and their cognitive impacts largely disappear within a few years” (p.110). Duncan and Magnuson do suggest that more recent studies suggest possible longer-term effects on years of education, earnings, and lower crime, but clearly the evidence is mixed on the effectiveness of early childhood schooling, a currently popular intervention. The evidence of fade out of effects suggests a possible major contribution for “brain approaches.” That is, since findings regarding short and longer-term impacts on “cognitive and noncognitive” outcomes are mixed, it is uncertain what investments in skills, behaviors, or developmental processes are particularly important in producing positive impacts across the child’s life span. The National Academies of Sciences, Engineering, and Medicine sponsored a 2019 report that responded to a Congressional mandate “to identify evidence-based programs and policies for reducing the number of children living in poverty in the United States by half within 10 years” (p.1). While they found evidence that a handful of programs (such as the earned income tax credit, the Supplemental Nutrition Assistance Program, and housing subsidies), do reduce poverty and lead to better child outcomes, the evidence of how best to spend public dollars remains limited. The report concludes: the “[Office of Management and Budget] should also convene working groups charged with assessing the report’s recommendations for research and data collection to fill important gaps in knowledge about effective anti-child-poverty programs” (p. 6). We believe brain-based research can help us to learn how best to spend public dollars in this endeavor.

## How Can these Data be Applied to Helping Disadvantaged Children?

Below, we suggest five ways in which neuroscience-based approaches can be harnessed to improve the circumstances of children living in poverty.

### Leverage a culture that values biology

Though perhaps the least scholarly benefit of neuroscience, this rationale may nonetheless confer significant benefit to anti-poverty efforts on behalf of children. For better and for worse, issues that are framed as biomedical tend to get attention, are elevated as priorities, and receive support that is not viewed as politically partisan. Furthermore, there is evidence that neuroscience data are viewed by the general public as especially compelling. For these reasons, bringing brain-based measures to bear upon issues of child poverty holds potential to not only to demonstrate effects of social programs, but to also increase the likelihood that these effects are noticed and discussed by policymakers.

This is such a nonscholarly argument that we want to be clear about what we are not saying. First, neuroscience data do not have elevated ontological status relative to behavioral evidence. Second, no one needs neuroscience data to convey that poverty is bad. Third, all behavior has a neurobiological underpinning, so the mere fact that a behavioral phenomenon has a brain correlate is hardly a groundbreaking insight. What is of potential value – aside from real advances in understanding how poverty influences basic aspects of children’s biological development – is that at the very least, studies that provide neurobiological evidence may bring more interested parties to the table. There is potential leverage to be gained from the fact that neuroscientific evidence is often assumed – incorrectly – by laypersons to be more valid and robust because the lay public often lacks the training or expertise that would enable them to view neuroscience data through a critical lens. The lack of knowledge that most laypersons have about the workings of the brain, much less the nuances of neuroscientific methods, often leads them to be overly impressed by brain science, even when behavioral research may be more relevant to policy decisions. The point is not that neuroscience data are not useful or are somehow “duping” the general public. We are simply stating that if brain data engage the interest and attention of people who might not otherwise be inclined to support anti-poverty programs, that is a real benefit for everyone.

There has been some empirical research about the extent to which neuroscience data compel people. Some studies report that brain images have a particularly persuasive influence and that explanations of psychological phenomena generate more public interest when they contain neuroscientific information; for example, presenting brain images with articles resulted in higher ratings of scientific rigor for arguments made in those articles as compared to articles accompanied by bar graphs (McCabe & Castel, 2008). Even irrelevant neuroscience information may influence how people judge scientific information: people judged study descriptions containing irrelevant neuroscience information as more satisfying than explanations without such data (Weisberg, Keil, Goodstein, Rawson, & Gray, 2008). One study even found that the effects of brain images on evaluations of scientific reports was moderated by the way those images were presented, with three-dimensional pictures of neuroimaging results producing more positive evaluations (Keehner, Mayberry, & Fischer, 2011). These data lend support to the notion that part of the fascination with neuroscience research lies in the persuasive power of the actual brain images themselves, which provides a seemingly physical basis for abstract processes, and appeals to people’s affinity for reductionistic explanations of complex phenomena, or at least piques a fascination with the idea of insight into the human brain (Beck, 2010).

Some scientists have questioned the idea that people are especially compelled by brain images, calling this is a “persistent meme” without empirical support (Michael, Newman, Vuorre, Cumming, & Garry, 2013). One study failed to replicate the earlier observations in this regard, finding no general evidence of a neuroimage bias in people’s evaluation of scientific reports. Yet this same study noted that when laypeople are exposed to multiple sources of data (e.g., when directly comparing neuroimages to other depictions of data), a limited neuroimage bias was observed (Schweitzer, Baker, & Risko, 2013). One possibility is that between 2008, when the original neuroimaging bias studies were conducted, and 2013, when these findings were questioned, the general public became less influenced by brain images. A more likely explanation is supported by a recent set of experiments evaluating whether neuroscience information, more broadly construed that just brain images, make explanations of psychological phenomena more appealing. This study was done while controlling for participants’ analytical thinking abilities, beliefs on free will, and admiration for science. The researchers found that accompanying functional MRI (fMRI) pictures had no impact above and beyond the neuroscience text, but that people found neuroscience information more alluring than both social science and physical science information. People’s analytical thinking did not protect against the neuroscience bias, nor did a belief in free will (Fernandez-Duque, Evans, Christian, & Hodges, 2015). Thus, the “allure of neuroscience” appears to be conceptual rather than merely pictorial, reflecting lay beliefs about the explanatory power of the human brain. In other words, the language and imprimatur of neuroscience itself is compelling.

Harnessing the power of this allure to heighten interest and concern about the effects of child poverty is a net positive, but it is not without risks. The general public may assume that biological correlates of some behavior demonstrate that the behavior cannot be changed and that an individual has some sort of permanent deficit. Such a conclusion would likely be false, given evidence that the brain is malleable, and there is a good deal of evidence that human brains have periods of heightened neuro-plasticity. Related to a confidence in all things biological is the common misunderstanding that something “biological” is somehow innate and not the result of environmental factors – a false conclusion belied by decades of empirical studies. In sum, conveying that children in poverty show less activation in a brain region or neural system can be extremely compelling to someone with little knowledge neuroscience, but also confers some risk of misunderstanding. Brain-based data sound both definitive and scientific, especially because in most cases, presentations to policymakers do not afford the time to explain the complicated processes of arriving at these conclusions. For this reason, policymakers may construe an fMRI image as a photograph, or akin to an X-ray image. In most brief interactions with policymakers, it may not be the best use of time to undertake an explanation of the fact that fMRI images are highly processed interactions between radio waves and the magnetic properties of hydrogen and deoxygenated hemoglobin. Perhaps ironically, the complexity of the neuroscience methods themselves may well lead laypeople to have greater confidence in the scientific rigor of the images than in the behavioral phenomena that initially motivated the neuroscience study. Thus, while it is useful for the public to be informed about ongoing research, this usually requires that complex methods and findings are distilled into a simple message; the difficult part is making sure that the simple message communicates what can and cannot be concluded from the data.

### Neural activity might reveal processes underlying disparities not otherwise observable, and that might also (hopefully) be responsive to change and generate new or more refined hypotheses

Although studies have been successful at documenting the range of negative sequelae associated with exposure to poverty in childhood, questions about the specificity and distinctiveness of the mental processes affected by these experiences have been elusive. There are a number of ways in which brain data might elucidate developmental mechanisms, or at least provide a physiological grounding to constrain or refine hypotheses regarding how and why economic deprivation affects child development. This is because in vivo human brain-related responses can provide a window into potential subcomponents of cognitive functioning, or mental processes generally, that may not be observable from overt behavior. This is achieved not by focusing only on “where” brain activation differences occur, but “how” the brain appears to be processing different kinds of information.

One education-relevant example is attention, a common but highly complex phenomenon with many distinct subcomponents. Attention often has the appearance of a unitary system, and it is not uncommon to hear children described as having generally good or poor attentional functioning. This tendency to generalize about attention may arise because many of the behavioral consequences of attention covary and are difficult to discern. However, in the brain, attention-related changes in neuronal activity are observed in widespread structures, suggesting that attention results from subcomponents corresponding to distinct biological mechanisms (Luo & Maunsell, 2019). It is possible that exposure to childhood poverty affects some particular neural systems, or that some of these systems might be most amenable to change. If so, knowledge of this processes would allow for more targeted – and perhaps more effective –interventions.

The notion that attention includes distinct components and forms is well established. To illustrate, one aspect of attention involves sustaining vigilance over a long time period to maintain performance across a task. Children need attentional vigilance to pay attention over the course of a lesson or class, during a story or presentation, or while reading. This is essentially preventing the mind (or eyes) from wandering and staying engaged for a set period of time. A different aspect of attention involves switching engagement, such as changing from one activity to another, or attending to what a teacher is doing in front of a classroom while also attending to the materials on one’s own desk. Working on an assignment while also monitoring the time left to complete the assignment is also example of this type of attention. Yet another aspect of attention involves selecting what in the environment is relevant and important, and dismissing irrelevant information so that cognitive resources are deployed to important stimuli. In this regard attention can be more of less selective. Further, attention is also subdivided according to what caused it to be deployed: physical events in the environment (such as verbal instructions or a loud noise) versus internal factors under voluntary control versus lingering effects of what someone has recently learned or experienced (Maunsell, 2015). Finally, attention can be still further subdivided into whether it is overt attention (associated with detectable behavior) or covert attention (when attention changes with no outward manifestation). This range of examples is meant only to highlight the many ways in which aspects of attention can span timescales, functions, and goals that are not easily separated through behavioral measures (Fortenbaugh, DeGutis, & Esterman, 2017).

Similar to attention, cognitive functions such as memory also have subcomponents that might be selectively impaired or remediated. It is now recognized that different components of memory depend on separate brain structures. For example, behavioral data could not reveal that separate processes underlie the abilities to recall something directly versus recognizing something as seeming familiar (Henson, 2005). Different subskills necessary for effective reading are associated with activation in separate brain regions (Welcome & Joanisse, 2012). Generally, in most behavioral tasks, it is difficult to manipulate or measure a component of a participant’s attention without also capturing other cognitive processes, such as reward expectation, motor preparation, or working memory.

Besides specific skills relevant to children’s healthy development, functional neuroimaging has potential to reveal the general processes through which early adverse experiences might affect children’s learning (Smith & Pollak, 2020). By indexing fluctuations of neural activity, neuroimaging allows for an examination of the processes through which children acquire new information or skills rather than a focus solely on the outcome of learning (Karuza, Emberson, & Aslin, 2014). As with attention, memory, and reading, learning is often referred to as a single process, but the concept subsumes many different operations and neural processes. Thus, there might be multiple neural and psychological processes that are differentially affected by the adversity associated with poverty. As just a few of many possibilities, there appear to be distinct and separable neural processes for acquiring new information as compared to using that information (McNealy et al., 2006), making predictions based upon learned information (Waelti, Dickinson, & Schultz, 2001), and learning the cause of an outcome (Gershman & Niv, 2010).

There are likely changes in neural activity *before* there can be behavioral evidence of learning, presumably during initial exposure to stimuli, before corresponding behavioral changes are evident. Thus, based upon overt behavior alone, it is difficult to differentiate participants who can learn, but do so more slowly versus those who experience difficulties at the earliest stages of learning. The time course of learning is a reliable and important individual difference (Turk-Browne, Scholl, Johnson, & Chun, 2010), and one that might be especially important in designing anti-poverty early childhood education programs. For example, some children might do well earlier in the learning process and then poorly later (something akin to fatigue), whereas other may have poor performance early on and see their performance improve later in the learning process (akin to needing a “warm-up”). The time course of learning, and the trajectory of learning during a task has emerged as an important variable in accounting for social and educational difficulties in children exposed to very high levels of adversity and stress (Hanson et al., 2017; Harms, Shannon Bowen, Hanson, & Pollak, 2018). These are insights that cannot be observed without methods that allow analyses of how children continue to process information after they are exposed to it (Karuza et al., 2014).

Our point here is not that brain measures are an ideal or even the best possible research approaches. They are among many tools and have their limitations. There are many examples of cases where brain activity is uninformative about the similarity of psychological tasks. For example, it is always possible that two tasks might involve the same brain regions but use different populations of neurons or different patterns of connectivity between regions. Conversely, two tasks might involve different regions but involve the same type of computation. Observed brain activation may not be essential for a given task at all. Overall, however, behavior alone might not have the specificity needed to effectively tailor interventions for at-risk children because many different theories about interactions between brain processes rely on similar behavioral predictions (White & Poldrack, 2013). For these reasons, insights into neural processes hold promise to help us understand questions such as when in development children are most vulnerable, when interventions may effect maximal change, which processes are amenable to remediation, and how much interventions are needed to effect change.

### Brain physiology may predict behavior better than available behavioral measures

fMRI is usually used in clinical research to show differences between groups. However, patterns of brain activity can prospectively predict important behavioral outcomes across a range of domains, with increasing evidence that neuroimaging data (and potentially other brain physiology measures) serves as a better predictor of future behavior than traditional behavioral measures such as self-reports, clinical rating scales, or scores on educational or neuropsychological tests (Gabrieli, Ghosh, & Whitfield-Gabrieli, 2015). Therefore, there is good reason to suggest that future studies might leverage neuromarkers for individualized predictions of educational or health outcomes for children living in poverty. Such data could be used to develop novel intervention strategies, or perhaps individually optimize the type of timing of educational and clinical practices for children most susceptible to poor outcomes.

There are three different ways that prediction can be useful for studies of child poverty. The first is the approach most often used in research. That is, prediction is used simply to refer to correlation between two contemporaneous values, such as a score on some task (such as a measure of impulsivity) being associated with some individual difference variable (such as regional brain activation). This type of study is useful in uncovering mechanisms underlying maladaptive behaviors. That is not, however, our primary focus here.

Prediction can also refer to within-sample changes over time. For example, task performance when a cohort is aged 5 years predicting an outcome when that cohort is aged 10 years. This is a very different kind of analysis from the first, and more common, use of prediction because significant group differences (detected via *t* tests) are more likely to occur when there is high within-group homogeneity. In contrast, factors associated with the likelihood of predicting future outcomes harness heterogeneity within a sample. Variables that significantly differentiate between groups are often weak predictors of future behavior (Lo, Chernoff, Zheng, & Lo, 2015). To explore the utility of this predictive approach, Jollans and Whelan (2016) reviewed studies that used neuroimaging measures to predict treatment response and disease outcomes in a range of psychiatric and neurological illnesses. They found that many of the studies were able to predict behavioral outcomes, with neuroimaging data often augmenting the prediction compared to clinical or psychometric data alone. Based upon their meta-analytic review, they report that brain measures explain a significant amount of variance where clinical and behavioral variables fail to do so, with brain measures accounting for up to 40% of the variance in clinical outcomes. Moreover, in a number of studies that Jollans and Whelan reviewed, it was only the neuroimaging variables that successfully predicted clinical outcomes. In this regard, although measures of brain physiology may be expensive or difficult to collect, the benefit may exceed the cost of unsuccessful interventions and educational failure for children.

A third way that neuroimaging data can be used holds tremendous promise for policy and intervention development. This approach involves predicting outcomes for *new* individuals based upon previously collected data from *other* individuals. In this case, prediction refers to a generalizable model; a study with a sample that is used to predict the behavior of individuals who were not part of that original sample (Berkman, Falk, 2013). In this manner, a relatively small (easily collected, less expensive) sample is used to make predictions or treatment decisions for a larger population (Falk, Berkman, & Lieberman, 2012). This may represent a powerful and feasible way to evaluate prevention and intervention programs for children in poverty.

Below we provide just a few illustrative examples of how neuroimaging has been used to make educationally or clinically useful predictions. We draw these samples from a range of different domains that are relevant to poverty studies.

#### Prediction of reading development

Neuroimaging measures have been shown to enhance and even outperform traditional behavioral measures in forecasting children’s reading abilities. In studies such as these, children are identified by their teachers as having reading problems and then evaluated with behavioral tests of reading and reading-related skills as well as fMRI tasks. One longitudinal study examined how the behavioral measures, fMRI activation for a word-rhyming task, and diffusion tensor imaging (DTI) indices of white matter organization predicted reading difficulties three grade years ahead (Hoeft et al., 2011). This study reported that none of the behavioral measures correlated with future reading gains, but the brain measures did. High levels of activation in the right prefrontal cortex (PFC) and white matter organization of the right superior longitudinal fasciculus predicted, with 72% accuracy, whether children’s reading problems persisted. In another longitudinal study, 9–15 year old children were initially assessed for reading skill and performed an fMRI rhyming judgment task. The patterns of brain activation in the fMRI task predicted the type of difficulties that children encountered in their reading 6 years later. Increased activity relative to peers in neural circuits associated with phonological recoding (i.e., inferior frontal gyrus and basal ganglia) predicted which children would show greater gains in reading fluency among the younger children, whereas increased activity relative to peers in orthographic processing circuits (i.e., fusiform gyrus) was predictive of smaller gains in fluency for older children (McNorgan, Alvarez, Bhullar, Gayda, & Booth, 2011). The results suggest that younger children who are more sensitive to phonological word characteristics make greater reading proficiency gains, whereas older children who focus more on whole-word orthographic representations make smaller proficiency gains. A third example is a study involving kindergarteners who were not yet reading. They were administered a combination of behavioral measures, event-related potentials (ERPs), and fMRI responses to presentations of printed letters; these measures, in combination, explained 88% of the variance in reading ability when those children reached second grade (Bach, Richardson, Brandeis, Martin, & Brem, 2013). These data suggest that neuromarkers can be used to identify children who will encounter difficulties learning to read even before reading instruction begins in school. As Gabrieli et al. (2015) point out, this is important because current reading interventions are most effective in young, beginning readers, and effective intervention prior to reading failure may not only be more effective but also spare children the sense of failure that often accompanies early struggles in reading. Using fMRI or ERPs in a predictive manner could also help tailor the kinds of educational interventions that may be most beneficial for individual children and are certainly amenable to cost–benefit types of analyses.

#### Prediction of substance abuse

In a longitudinal study, 12- to 14-year-olds with little or no history of substance abuse performed a go/no-go task of response inhibition while undergoing fMRI (Norman et al., 2011). About 4 years later, those fMRI results accurately predicted those adolescents who did or did not transition to heavy use of alcohol. Reductions in activation within the prefrontal and anterior cingulate cortices predicted adolescents who later transitioned to heavy alcohol use relative to those who did not. A separate study reported highly convergent results. Among adolescents 16–19 years of age with an ongoing history of substance use disorders, those who exhibited less prefrontal and greater parietal activation on a similar go/no-go the task had higher levels of substance use 18 months after scanning (Mahmood et al., 2013).

#### Prediction of depression

fMRI data have successfully predicted disease course in patients with depression. One study reported that clinical variables, such as the number of previous depressive episodes, depression symptom severity, and time in remission, did not alone predict whether patients remained in remission after 14 months. However, outcome predictions reached 75% accuracy on the basis of fMRI data gathered during a self-versus other-blaming task (Lythe et al., 2015). Another study found that activation in the subgenual anterior cingulate cortex during an emotion information processing task measured prior to treatment predicted which depressed patients had the most improvement following a cognitive behavioral treatment (Siegle et al., 2012). A similar study found the same pattern of results – and it is noteworthy that only the brain physiology variables successfully predicted clinical outcomes (Gong et al., 2011). Thus, brain physiology appears to provide a clinically applicable way of assessing neural systems associated with treatment response.

#### Prediction of healthy eating

Healthy eating to avoid or reduce obesity is also a major public health concern. Neuroimaging studies have reported that fMRI activations in response to food-related pictures forecast future changes in body mass index (BMI). One study examined the relation between baseline fMRI activations and weight gain over the following year in adolescent females ranging from lean to obese using an attention task involving food and neutral stimuli. fMRI measures of activation in brain regions including the anterior insula/frontal operculum, lateral orbitofrontal cortex (OFC), ventrolateral prefrontal cortex (vlPFC), and superior parietal lobe correlated with future increases in BMI 1-year later (Yokum, Ng, & Stice, 2011). These are networks tied to attention and reward processing. None of the behavioral measures predicted future weight gain. Many people who engage in weight loss interventions fail to reach targeted goals or maintain their efforts. A recent study used neuroimaging to predict success in healthy eating based upon the idea of identifying individuals most amenable to behavioral change. fMRI data were prospectively collected prior to a behavioral weight loss intervention involving overweight adults. Machine learning and functional brain networks predicted which adults would continue to follow through with the intervention 18 months later with 95% accuracy (Mokhtari et al., 2018). Connectivity patterns that contributed to the prediction consisted of brain networks that are associated with self-regulation, body awareness, and the sensory features of food.

In sum, the potential to tailor personalized treatment plans or early interventions, to identify individuals in need of most intensive interventions, or to identify larger populations of individuals who can benefit from a treatment based upon smaller samples could have considerable implications for the economic cost of health care and educational practice. Of course, it is reasonable to consider whether using brain measures as part of educational planning is practical given the costs and need for children to visit imaging facilities. As noted above, cost–benefit analyses would help determine whether brain physiology techniques are appropriate, justified, and feasible. Other less costly indices of brain function, such as ERPs, may also be useful in this regard. ERPs are far less costly and the equipment is relatively easily transported to school and community settings. However, given the long-range costs of educational deficits, and the nonnegligible costs of traditional psychological and educational assessments and of interventions with modest efficacies, such an option may pass a cost–benefit test, especially for severely delayed learners.

### Neural measures may allow us to evaluate interventions and policies earlier. (including “shadow effects”)

When a new program or intervention is introduced, even with an experimental design, the evaluation of that program is usually focused on relatively short-term outcomes. Rarely are studies designed (or funded) to measure long-term outcomes such as future earnings of children enrolled in a preschool program. Researchers and funders are rarely willing to wait for a decade or more to measure potential outcomes. Today, it is possible to use administrative records (along with consent agreements) to gather such data, but researchers must still wait a long time to access such data, and participant attrition may reduce reliability. One example of the importance of measuring longer-terms outcomes associated with an intervention (also called shadow effects) is the Moving to Opportunity (MTO) study. MTO is an experimental program that offered some families the opportunity to move out of high-poverty neighborhoods (Chetty, Hendren, & Katz, 2016). Earlier research had found that this housing experiment had only small effects on children, and it appeared that these effects faded over time. However, more recent examination of the data revealed shadow effects that were not apparent until the children were older. Among the positive effects associated with children’s families moving to low-poverty neighborhoods before the children were 13 years of age included an increased probability of attending college and higher earnings during their mid-20s. This result did not hold for children who moved when they were older. The study used tax data to discover the longer-term results. For this reason, these effects of the intervention could not be seen when children were young, but only after they had entered the workforce. It is possible that the use of neuroscience approaches might allow us to capture early signs of these long-run outcomes, and thereby identify programs that are likely to be effective.

### Bootstrapping extant neuroscience knowledge

We currently understand little about how and why poverty can have such devastating effects on children’s healthy development, but the neuroscience literature provides some insight into factors that may serve as causal mechanisms linking poverty to poor health and educational outcomes. Therefore, we can draw from literatures on the effects of extreme stress and adversity, ranging from exposure to toxins to nutritional restriction to housing and food instability to limited family resources to dangerous neighborhoods to parental stress. These studies of various forms of stress can provide insight into the mechanisms that may affect children living in poverty.

There are consistent relationships between high levels of stress exposure and disruption of the hypothalamic–pituitary–adrenal (HPA) axis (Koss & Gunnar, 2017; Strüber, Strüber, & Roth, 2014), autonomic nervous system (Esposito, Koss, Donzella, & Gunnar, 2016), and immune system functioning (Danese & Lewis, 2017; Danese, this issue; Miller & Chen, 2010; Müller et al., 2019), as well as epigenetic changes, especially in the glucocorticoid receptor gene (Papale, Seltzer, Madrid, Pollak, & Alisch, 2018; Romens, McDonald, Svaren, & Pollak, 2015; Turecki & Meaney, 2016; Tyrka, Price, Marsit, Walters, & Carpenter, 2012). These are systems that have implications for issues of behavioral regulation, academic performance, and health. A recent, and important, longitudinal study demonstrates that such effects on the HPA system remain open to recalibration in humans if environmental factors improve (Gunnar, DePasquale, Reid, & Donzella, 2019). This suggests that anti-poverty intervention efforts should include a focus on the prepubertal and peripubertal period in order to maximize their impact on recalibrating systems like the HPA axis.

In addition, physiological alterations in the stress system appear to be linked to functional and structural changes in a number of brain regions (Fan et al., 2014; Gorka, Hanson, Radtke, & Hariri, 2014; Palacios-Barrios & Hanson, 2019; Tottenham & Sheridan, 2009). To illustrate, chronic stress is associated with global changes in dendritic branching and synaptic plasticity throughout the PFC, amygdala, and hippocampus – circuitry that has been implicated in alterations in learning, memory, and stress responsivity (Hostinar & Gunnar, 2013; Ironside, Kumar, Kang, & Pizzagalli, 2018; McEwen & McEwen, 2017; Novick et al., 2018). All of these domains have arisen in descriptions of outcomes associated with child poverty. Recent studies suggest that early adversity may lead to altered connectivity between the amygdala and PFC (Gee et al., 2013; VanTieghem & Tottenham, 2018). Comparable alterations in development of the hippocampus are observed in children who experienced a variety of experiences including abuse, neglect, poverty, and general chronic stress (Gorka et al., 2014; Hanson et al., 2015; Teicher et al., 2018). These stress-related changes all appear to be similarly, and at least partially, mediated by corticotropin-releasing hormone (CRH) and glucocorticoids, key regulators of the HPA axis (Koss & Gunnar, 2017; McEwen & Morrison, 2013; Vazquez et al., 2006; Wang et al., 2011).

There are some other promising lines of research on the neurobiology of stress that are highly relevant to the experiences of children living in poverty. We highlight some of these briefly below.

#### Perceptions of insecurity

Children’s perceptions of scarcity or insecurity associated with family poverty might influence their neurobiology (Brosschot, Verkuil, & Thayer, 2017; Lazarus & Folkman, 1984; McEwen, 2019; Peters, McEwen, & Friston, 2017; Sapolsky, 2015). This type of effect depends upon how organisms perceive the controllability and predictability of stressors (Bollini, Walker, Hamann, & Kestler, 2004; Muller, 2012). In humans, individual differences in perceptions of control have been linked to differential cortisol responses to acute laboratory stress, differences in brain volume, and differences in brain reactivity to stress in regions including the hippocampus, amygdala, and PFC (Harnett et al., 2015; Hashimoto et al., 2015). This may be a critical factor in cases where housing and food are insecure.

Yet, it is not simply the case that how a potential stressor is perceived attenuates or exacerbates physiological responses. Rather, individual’s perceptions of their own circumstances trigger different patterns of responses across neural systems. As an illustrative example, if individuals construe their personal resources as sufficient to outweigh a situational demand, they evince increased sympathetic cardiac activation, accompanied by increased cardiac output and decreased vascular resistance. In contrast, if individuals perceive that same situation as outweighing their personal resources, their increased sympathetic cardiac activity is accompanied by decreased cardiac efficiency, including changes in cardiac output and increased vascular resistance (Mendes & Park, 2014; Sammy et al., 2017). These cardiovascular patterns have been linked to distinct patterns of HPA activation (Seery, 2011). This basic science has clear applicability to individuals developing within under-resourced environments. Other factors that influence how individuals interpret potential stressors include whether individuals perceive themselves to be in a safe or dangerous environment (Blascovich, 2008; Jamieson, Hangen, Lee, & Yeager, 2018), which may account for the effects of children living in dangerous or loud neighborhoods.

#### Intensity and cumulative stress

Humans also evince increases in sympathetic noradrenergic, adrenomedullary, and HPA responses for a range of stressors that vary according to the intensity of the stressor (Ouellet-Morin et al., 2019), and stressors perceived as more intense are associated with larger cortisol responses (Skoluda et al., 2015). For this reason, it is important to remember that the context of poverty does not involve any single stressor for children, but a wide net of different sources of stress over protracted periods of time. One study reported that children with high levels of chronic life stress had smaller amygdala and hippocampal volumes than children exposed to less intense levels of early adversity (Hanson et al., 2015). Germaine to this discussion, it is noteworthy that children with reports of child abuse, neglect, and those living in poverty all showed similar effects on brain structure, suggesting a common stress-related mechanism across these early life experiences. Yet another study found that individuals who experienced high levels of adversity when they were children demonstrated altered activation in circuits involved in risk taking and decision making when they were young adults (Birn et al., 2017). These effects were not explained by the stress in the participant’s current adult lives, but only their childhood experiences.

#### Environmental instability

Recent research also suggests that predictability in the environment shapes children’s cognitive outcomes (Davis et al., 2017; also Davis, this issue). Longitudinal research finds that unpredictability, including factors highly relevant to poverty such as frequent changes in maternal employment, residence, and cohabitation, was associated with increased externalizing behaviors in adolescence (Doom, Vanzomeren-Dohm, & Simpson, 2016). Similarly, research in rodents indicates that these observed effects are a result of altered functioning in prefrontal–hippocampal–amygdala circuits, finding that lack of stability in the early environment is associated with altered connectivity between the medial PFC and amygdala (Bolton et al., 2018), as well as decreased dendritic arborization in the hippocampus (Molet et al., 2016). Together, this body of work is consistent with the view that better assessment of variation in the predictability, stability, and/or degree of contingent responding of adult caregivers to the needs of the developing child will provide insight into developmental alterations in prefrontal cortical and subcortical stress response circuits (for discussion, see Smith and Pollak, In press).

In sum, there is not likely to be a brain signature specific to poverty. For one, the experience of poverty involves many different kinds of experiences converging on children and their families over time. In addition however, the brain is unlikely to respond in distinct ways to the variety of adversities that humans might encounter. For these reasons, it will be productive to apply and built from the extant body of knowledge about the neurobiology of stress to further our understanding of the effects of family poverty on children’s development.

## Conclusion

Child poverty represents a worldwide humanitarian, public health, and pragmatic problem. Poverty affects the lives of millions of children and needs more progress and new ideas based upon a variety of scientific evidence. From a humanitarian perspective, poverty represents not merely low income, but a deprivation of children’s human capabilities (Sen, 1985). From an economic perspective, the cost of poverty is high. For example, problems associated with poverty, including child maltreatment, crime and incarceration, reduced earnings, health problems, and child homelessness cost the United States $1.03 trillion dollars in 2016 (McLaughlin & Rank, 2018). The number represented 28% of the entire federal budget that year. The impact of most early childhood anti-poverty programs is, however, quite modest (Duncan & Magnuson, 2011). We know that impoverished children are likely to grow up with fewer skills to contribute to society because of educational under-attainment and are disproportionately likely to experience more serious health problems. These costs are borne by the children themselves, but by the wider society as well.

For these reasons, neuroscientific approaches may be successfully married with social science approaches to generate new clues about possible prevention and intervention policies and programs. It is not that there will be a clear brain signal that is diagnostic of poverty, or that any single neural process affected by poverty will be a direct cause of poor outcomes among impoverished children. Poverty represents many different kinds of social interactions, challenges, and stressors over the course of a child’s development; but developmental neuroscience does have a rich corpus of data that can help.

At the same time, the use of neuroscience to better understand poverty must be undertaken in a way that is mindful of three important issues. The first is the fact that most neuroscience techniques, such as fMRI and ERPs, are well suited to questions with few variables that can be examined with a limited range of response options. However, the effects of poverty likely involve very complex problems with multiple variables. The second is that the brain is unlikely to be wired in a way that specifically responds to different aspects of the wide variety of possible human experiences. Therefore, brain effects are likely not going to be specific to poverty, per se, but to generalize broadly to the effects of chronic adversity on child development.

The third is that biological correlates of poverty may represent powerful opportunities for policy, but the biology-policy links will be nonobvious or direct. In general, policymakers care about broad social metrics such as improving health or mortality rates, increasing high school graduation rates, or positive employment outcomes. Developmental psychologists study constructs such as executive functions, self-regulation, and phonemic awareness; neuroscientists study phenomena such as brain connectivity, hippocampal volume, hormone fluctuations, and synapses. Simple solutions should not be expected, and simple causal explanations perhaps viewed with skepticism. However, with thoughtful, integrative and cross-disciplinary work, linking these levels of analyses shows great promise for targeting and refining new and effective interventions, programs, and policies. We place great hope on using new ways to combine scientific tools and multidisciplinary insights to ensure equity in children’s health, success, and well-being.
